# 3,28-Diacet­oxy-29-bromo­betulin

**DOI:** 10.1107/S1600536809027640

**Published:** 2009-07-25

**Authors:** Wei-Min Ding, Li-Jia Jing, Tao Yu, Yang Wang, Xiu-Feng Yan

**Affiliations:** a College of Life Sciences, Northeast Forestry University, Harbin 150040, People’s Republic of China; b School of Chemical and Environmental Engineering, Harbin University of Science and Technology, Harbin 150080, People’s Republic of China

## Abstract

In the title mol­ecule, C_34_H_53_BrO_4_, all the cyclo­hexane rings adopt chair conformations, while the cyclo­pentane ring adopts an envelope conformation. In the crystal, weak inter­molecular C—H⋯O hydrogen bonds link the mol­ecules into corrugated sheets parallel to the *ab* plane.

## Related literature

For the anti-HIV and anti­tumor activities of betulin derivatives, see: Sun *et al.* (1998 ▶) and Kim *et al.* (1998 ▶), respectively. For a related structure, see Mohamed *et al.* (2006 ▶).
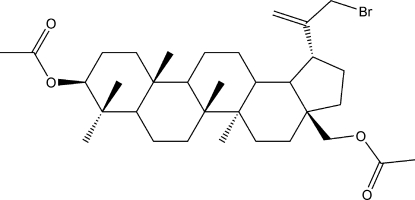


## Experimental

### 

#### Crystal data

C_34_H_53_BrO_4_
*M* *_r_* = 605.67
Orthorhombic, 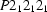

*a* = 7.152 (3) Å
*b* = 14.946 (7) Å
*c* = 29.837 (12) Å
*V* = 3189 (2) Å^3^
*Z* = 4
Mo *K*α radiation
μ = 1.32 mm^−1^
*T* = 291 K
0.40 × 0.38 × 0.37 mm

#### Data collection

Rigaku R-AXIS RAPID diffractometer
Absorption correction: multi-scan (*ABSCOR*; Higashi, 1995 ▶) *T*_min_ = 0.622, *T*_max_ = 0.642
23319 measured reflections
5578 independent reflections
3534 reflections with *I* > 2σ(*I*)
*R*_int_ = 0.102

#### Refinement

*R*[*F* ^2^ > 2σ(*F* ^2^)] = 0.057
*wR*(*F* ^2^) = 0.145
*S* = 0.94
5578 reflections
358 parameters
18 restraints
H-atom parameters constrained
Δρ_max_ = 0.45 e Å^−3^
Δρ_min_ = −0.58 e Å^−3^
Absolute structure: Flack (1983 ▶), 2346 Friedel pairs
Flack parameter: 0.024 (12)

### 

Data collection: *RAPID-AUTO* (Rigaku, 1998 ▶); cell refinement: *RAPID-AUTO*; data reduction: *CrystalStructure* (Rigaku/MSC, 2002 ▶); program(s) used to solve structure: *SHELXS97* (Sheldrick, 2008 ▶); program(s) used to refine structure: *SHELXL97* (Sheldrick, 2008 ▶); molecular graphics: *SHELXTL* (Sheldrick, 2008 ▶); software used to prepare material for publication: *SHELXL97*.

## Supplementary Material

Crystal structure: contains datablocks I, global. DOI: 10.1107/S1600536809027640/cv2584sup1.cif

Structure factors: contains datablocks I. DOI: 10.1107/S1600536809027640/cv2584Isup2.hkl

Additional supplementary materials:  crystallographic information; 3D view; checkCIF report

## Figures and Tables

**Table 1:** Hydrogen-bond geometry (Å, °)

*D*—H⋯*A*	*D*—H	H⋯*A*	*D*⋯*A*	*D*—H⋯*A*
C32—H32*A*⋯O4^i^	0.96	2.48	3.365 (7)	154
C28—H28*B*⋯O4^ii^	0.97	2.57	3.487 (6)	158

Symmetry codes: (i) 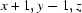; (ii) 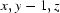.

**Fig. 1.**
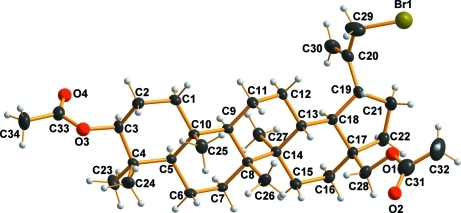
The molecular structure of (I) showing the atomic numbering and 50% probability displacement ellipsoids.

**Fig. 2.**
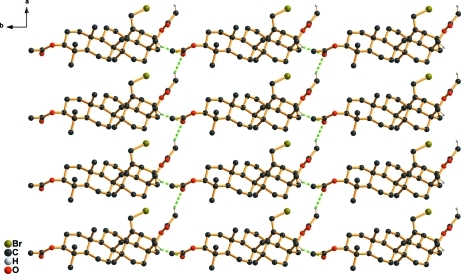
A portion of the crystal, showing the two-dimensional sheet of hydrogen-bonded (dashed lines) molecules. H atoms not involved in hydrogen bonds have been omitted.

## supplementary crystallographic information

### Comment

Betulin and its derivatives have been attracting extensive interests, owing to their anti-HIV and antitumor activites (Sun *et al.*, 1998; Kim *et al.*, 1998). The crystal structure of the diacetylation of betulin has been reported, considering the significance of its stereochemistry study. (Mohamed *et al.*, 2006). We report here the synthesis and the crystal structure of the title compound (I) - a new betulin derivative.

In (I) (Fig. 1), the cyclopentane ring adopts a twisted envelope conformation and all cyclohexane rings adopt chair conformations. The bond distances and angles are all within the expected ranges and agree with those in the similar compound reported previously (Mohamed *et al.*, 2006).

In the crystal, weak intermolecular C—H···O hydrogen bonds (Table 1) link the molecules into corrugated sheets parallel to *ab* plane.

### Experimental

Purified betulin (4.4 g, 10 mmol) was dissolved in dichloromethane (100 ml) and pyridine (0.5 ml) mixed solvent, followed by the addition of acetic anhydride (5 ml, 5.3 mmol). The reaction mixture was stirred for 24 h at room temperature. The solvent was removed by distillation under vacuum. The crude product was washed with a small quantity of benzene and then recrystallized from benzene (30 ml), 3.1 g diacetate-betulin was obtained.

The 3, 28-diacetate-betulin (1.0 g, 2 mmol), *N*-bromosuccinimide (0.35 g, 2 mmol) and benzoyl peroxide (0.05 g, 0.22 mmol) were dissolved in tetrachloromethane (50 ml). The reaction mixture was stirred for 6 h at reflux temperature. The solvent was removed by distillation under vacuum. The crude product was washed with a small quantity of ethanol and then recrystallized from petroleum ether (6 ml), 0.4 g suitable for X-ray diffraction test colourless block crystals of the titel compound was obtained.

### Refinement

C-bound H-atoms were placed in calculated positions and treated as riding on their parent atoms, with C—H = 0.96-0.98 Å and *U*_iso_(H) = 1.2-1.5Ueq(C).

### Crystal data

**Table:**

C_34_H_53_BrO_4_	*F*(000) = 1296
*M**_r_* = 605.67	*D*_x_ = 1.261 Mg m^−^^3^
Orthorhombic, *P*2_1_2_1_2_1_	Mo *K*α radiation, λ = 0.71073 Å
Hall symbol: P 2ac 2ab	Cell parameters from 15343 reflections
*a* = 7.152 (3) Å	θ = 3.1–27.4°
*b* = 14.946 (7) Å	µ = 1.32 mm^−^^1^
*c* = 29.837 (12) Å	*T* = 291 K
*V* = 3189 (2) Å^3^	Block, colourless
*Z* = 4	0.40 × 0.38 × 0.37 mm

### Data collection

**Table:**

Rigaku R-AXIS RAPID diffractometer	5578 independent reflections
Radiation source: fine-focus sealed tube	3534 reflections with *I* > 2σ(*I*)
graphite	*R*_int_ = 0.102
ω scans	θ_max_ = 25.0°, θ_min_ = 3.1°
Absorption correction: multi-scan (*ABSCOR*; Higashi, 1995)	*h* = −8→8
*T*_min_ = 0.622, *T*_max_ = 0.642	*k* = −17→17
23319 measured reflections	*l* = −35→35

### Refinement

**Table:**

Refinement on *F*^2^	Secondary atom site location: difference Fourier map
Least-squares matrix: full	Hydrogen site location: inferred from neighbouring sites
*R*[*F*^2^ > 2σ(*F*^2^)] = 0.057	H-atom parameters constrained
*wR*(*F*^2^) = 0.145	*w* = 1/[σ^2^(*F*_o_^2^) + (0.066*P*)^2^] where *P* = (*F*_o_^2^ + 2*F*_c_^2^)/3
*S* = 0.94	(Δ/σ)_max_ < 0.001
5578 reflections	Δρ_max_ = 0.45 e Å^−^^3^
358 parameters	Δρ_min_ = −0.58 e Å^−^^3^
18 restraints	Absolute structure: Flack (1983), 2346 Friedel pairs
Primary atom site location: structure-invariant direct methods	Flack parameter: 0.024 (12)

### Special details

**Table:**

Geometry. All e.s.d.'s (except the e.s.d. in the dihedral angle between two l.s. planes) are estimated using the full covariance matrix. The cell e.s.d.'s are taken into account individually in the estimation of e.s.d.'s in distances, angles and torsion angles; correlations between e.s.d.'s in cell parameters are only used when they are defined by crystal symmetry. An approximate (isotropic) treatment of cell e.s.d.'s is used for estimating e.s.d.'s involving l.s. planes.
Refinement. Refinement of *F*^2^ against ALL reflections. The weighted *R*-factor *wR* and goodness of fit *S* are based on *F*^2^, conventional *R*-factors *R* are based on *F*, with *F* set to zero for negative *F*^2^. The threshold expression of *F*^2^ > σ(*F*^2^) is used only for calculating *R*-factors(gt) *etc*. and is not relevant to the choice of reflections for refinement. *R*-factors based on *F*^2^ are statistically about twice as large as those based on *F*, and *R*- factors based on ALL data will be even larger.

### Fractional atomic coordinates and isotropic or equivalent isotropic displacement parameters (Å^2^)

**Table:**

	*x*	*y*	*z*	*U*_iso_*/*U*_eq_	
Br1	1.44746 (8)	−0.01367 (6)	0.18762 (2)	0.1130 (4)	
C1	1.0979 (5)	0.4597 (3)	0.38899 (13)	0.0394 (10)	
H1A	1.2251	0.4468	0.3798	0.047*	
H1B	1.0239	0.4685	0.3621	0.047*	
C2	1.0967 (5)	0.5466 (3)	0.41652 (14)	0.0419 (10)	
H2A	1.1780	0.5401	0.4423	0.050*	
H2B	1.1437	0.5955	0.3984	0.050*	
C3	0.9011 (6)	0.5672 (3)	0.43183 (13)	0.0384 (10)	
H3	0.8215	0.5744	0.4053	0.046*	
C4	0.8156 (5)	0.4940 (3)	0.46193 (12)	0.0366 (9)	
C5	0.8272 (5)	0.4054 (3)	0.43421 (12)	0.0311 (9)	
H5	0.7495	0.4169	0.4078	0.037*	
C6	0.7341 (6)	0.3239 (3)	0.45702 (14)	0.0430 (11)	
H6A	0.8162	0.3006	0.4801	0.052*	
H6B	0.6182	0.3424	0.4712	0.052*	
C7	0.6935 (5)	0.2506 (3)	0.42254 (13)	0.0380 (10)	
H7A	0.6044	0.2733	0.4008	0.046*	
H7B	0.6360	0.2002	0.4377	0.046*	
C8	0.8677 (5)	0.2177 (3)	0.39760 (12)	0.0308 (9)	
C9	0.9820 (4)	0.3005 (3)	0.37983 (11)	0.0294 (8)	
H9	0.9009	0.3276	0.3571	0.035*	
C10	1.0203 (4)	0.3785 (3)	0.41474 (11)	0.0281 (8)	
C11	1.1536 (5)	0.2692 (3)	0.35383 (13)	0.0379 (10)	
H11A	1.2395	0.2404	0.3744	0.045*	
H11B	1.2164	0.3211	0.3413	0.045*	
C12	1.1069 (5)	0.2039 (3)	0.31566 (13)	0.0367 (9)	
H12A	1.0373	0.2354	0.2926	0.044*	
H12B	1.2223	0.1825	0.3024	0.044*	
C13	0.9924 (5)	0.1240 (3)	0.33177 (12)	0.0310 (9)	
H13	1.0681	0.0934	0.3544	0.037*	
C14	0.8095 (5)	0.1562 (3)	0.35600 (12)	0.0324 (9)	
C15	0.6922 (6)	0.0744 (3)	0.37207 (14)	0.0445 (11)	
H15A	0.7519	0.0492	0.3984	0.053*	
H15B	0.5695	0.0955	0.3810	0.053*	
C16	0.6672 (6)	−0.0005 (3)	0.33708 (15)	0.0503 (11)	
H16A	0.6061	−0.0512	0.3511	0.060*	
H16B	0.5865	0.0208	0.3132	0.060*	
C17	0.8514 (6)	−0.0310 (3)	0.31707 (13)	0.0423 (10)	
C18	0.9435 (5)	0.0536 (3)	0.29593 (12)	0.0341 (9)	
H18	0.8472	0.0810	0.2770	0.041*	
C19	1.0936 (5)	0.0189 (3)	0.26382 (12)	0.0395 (9)	
H19	1.2080	0.0073	0.2809	0.047*	
C20	1.1404 (6)	0.0818 (3)	0.22529 (15)	0.0500 (12)	
C21	1.0121 (7)	−0.0725 (3)	0.24745 (15)	0.0582 (13)	
H21A	0.9779	−0.0688	0.2160	0.070*	
H21B	1.1044	−0.1195	0.2509	0.070*	
C22	0.8384 (7)	−0.0930 (3)	0.27615 (16)	0.0549 (12)	
H22A	0.7249	−0.0811	0.2594	0.066*	
H22B	0.8381	−0.1552	0.2854	0.066*	
C23	0.6078 (6)	0.5185 (3)	0.46879 (16)	0.0609 (14)	
H23A	0.5525	0.4779	0.4899	0.091*	
H23B	0.5431	0.5144	0.4407	0.091*	
H23C	0.5987	0.5785	0.4801	0.091*	
C24	0.9094 (6)	0.4905 (3)	0.50812 (12)	0.0520 (11)	
H24A	1.0428	0.4891	0.5045	0.078*	
H24B	0.8693	0.4377	0.5237	0.078*	
H24C	0.8749	0.5426	0.5251	0.078*	
C25	1.1663 (5)	0.3518 (3)	0.45069 (14)	0.0448 (11)	
H25A	1.2627	0.3163	0.4371	0.067*	
H25B	1.1063	0.3177	0.4739	0.067*	
H25C	1.2205	0.4048	0.4634	0.067*	
C26	0.9830 (6)	0.1609 (3)	0.43158 (12)	0.0464 (11)	
H26A	0.9780	0.1885	0.4606	0.070*	
H26B	1.1106	0.1575	0.4218	0.070*	
H26C	0.9314	0.1017	0.4333	0.070*	
C27	0.6862 (5)	0.2109 (3)	0.32242 (13)	0.0416 (10)	
H27A	0.6494	0.1730	0.2980	0.062*	
H27B	0.7568	0.2606	0.3111	0.062*	
H27C	0.5768	0.2325	0.3376	0.062*	
C28	0.9630 (7)	−0.0780 (3)	0.35488 (15)	0.0532 (12)	
H28A	1.0021	−0.0341	0.3769	0.064*	
H28B	0.8833	−0.1213	0.3698	0.064*	
C29	1.3386 (8)	0.0938 (4)	0.21333 (19)	0.0841 (19)	
H29A	1.4084	0.1103	0.2399	0.101*	
H29B	1.3494	0.1424	0.1919	0.101*	
C30	1.0135 (8)	0.1247 (4)	0.20139 (15)	0.0696 (16)	
H30A	1.0504	0.1613	0.1778	0.084*	
H30B	0.8873	0.1184	0.2082	0.084*	
C31	1.2306 (8)	−0.1665 (4)	0.3652 (2)	0.0732 (16)	
C32	1.3789 (7)	−0.2221 (4)	0.3428 (2)	0.094 (2)	
H32A	1.4944	−0.2161	0.3589	0.141*	
H32B	1.3957	−0.2019	0.3126	0.141*	
H32C	1.3411	−0.2837	0.3427	0.141*	
C33	0.8263 (7)	0.7245 (3)	0.43739 (18)	0.0534 (12)	
C34	0.8185 (9)	0.8011 (3)	0.46859 (19)	0.0819 (18)	
H34A	0.9413	0.8261	0.4719	0.123*	
H34B	0.7738	0.7812	0.4972	0.123*	
H34C	0.7354	0.8458	0.4569	0.123*	
O1	1.1239 (5)	−0.1224 (2)	0.33705 (11)	0.0625 (9)	
O2	1.2042 (7)	−0.1640 (4)	0.40533 (18)	0.1280 (19)	
O3	0.8996 (4)	0.65128 (19)	0.45704 (10)	0.0473 (7)	
O4	0.7726 (6)	0.7259 (2)	0.39882 (13)	0.0783 (11)	

### Atomic displacement parameters (Å^2^)

**Table:**

	*U*^11^	*U*^22^	*U*^33^	*U*^12^	*U*^13^	*U*^23^
Br1	0.0718 (4)	0.1855 (9)	0.0816 (4)	0.0435 (5)	0.0114 (3)	−0.0252 (5)
C1	0.039 (2)	0.044 (3)	0.035 (2)	−0.0083 (19)	0.0101 (17)	−0.0059 (19)
C2	0.047 (2)	0.037 (3)	0.042 (2)	−0.010 (2)	0.0035 (19)	−0.0031 (19)
C3	0.050 (2)	0.032 (2)	0.033 (2)	0.0026 (19)	−0.0026 (18)	−0.0114 (18)
C4	0.0410 (19)	0.038 (2)	0.0307 (19)	0.001 (2)	0.0084 (16)	−0.0040 (19)
C5	0.0329 (19)	0.032 (2)	0.029 (2)	−0.0004 (17)	0.0074 (16)	0.0006 (17)
C6	0.046 (2)	0.042 (3)	0.041 (2)	−0.008 (2)	0.0186 (19)	−0.001 (2)
C7	0.044 (2)	0.033 (2)	0.038 (2)	−0.0085 (19)	0.0154 (18)	0.0018 (19)
C8	0.0327 (18)	0.031 (2)	0.0285 (19)	−0.0018 (17)	0.0032 (16)	0.0014 (17)
C9	0.0260 (17)	0.036 (2)	0.0257 (18)	0.0005 (16)	0.0028 (14)	0.0018 (16)
C10	0.0263 (18)	0.033 (2)	0.0250 (18)	−0.0032 (16)	−0.0006 (15)	0.0006 (17)
C11	0.0321 (19)	0.038 (2)	0.044 (2)	−0.0041 (19)	0.0077 (17)	−0.0048 (19)
C12	0.0346 (19)	0.034 (2)	0.041 (2)	−0.0028 (17)	0.0098 (18)	−0.0103 (19)
C13	0.0304 (18)	0.031 (2)	0.032 (2)	0.0034 (16)	−0.0028 (15)	0.0021 (17)
C14	0.0334 (18)	0.031 (2)	0.032 (2)	−0.0026 (18)	0.0030 (16)	−0.0023 (17)
C15	0.046 (2)	0.039 (3)	0.048 (3)	−0.005 (2)	0.013 (2)	−0.006 (2)
C16	0.056 (2)	0.037 (3)	0.058 (3)	−0.016 (2)	0.008 (2)	−0.008 (2)
C17	0.055 (2)	0.031 (2)	0.041 (2)	−0.002 (2)	0.0046 (19)	−0.004 (2)
C18	0.0378 (18)	0.034 (2)	0.0303 (18)	0.0008 (18)	−0.0003 (16)	−0.0001 (17)
C19	0.050 (2)	0.037 (2)	0.0312 (19)	0.004 (2)	0.0009 (16)	−0.0066 (18)
C20	0.060 (3)	0.049 (3)	0.041 (2)	−0.003 (2)	0.010 (2)	−0.012 (2)
C21	0.083 (3)	0.045 (3)	0.047 (3)	−0.004 (3)	0.009 (2)	−0.012 (2)
C22	0.069 (3)	0.038 (3)	0.058 (3)	−0.007 (2)	0.001 (2)	−0.007 (2)
C23	0.049 (2)	0.052 (3)	0.082 (3)	−0.001 (2)	0.024 (2)	−0.021 (3)
C24	0.078 (3)	0.049 (3)	0.029 (2)	−0.007 (3)	0.0065 (19)	−0.004 (2)
C25	0.042 (2)	0.047 (3)	0.045 (2)	0.006 (2)	−0.0109 (19)	−0.005 (2)
C26	0.066 (3)	0.041 (3)	0.033 (2)	0.001 (2)	−0.008 (2)	0.007 (2)
C27	0.0335 (19)	0.051 (3)	0.041 (2)	0.0097 (19)	−0.0099 (17)	−0.004 (2)
C28	0.072 (3)	0.036 (3)	0.052 (3)	0.003 (3)	0.003 (2)	0.007 (2)
C29	0.075 (3)	0.115 (5)	0.062 (3)	−0.033 (4)	0.011 (3)	−0.009 (4)
C30	0.089 (4)	0.078 (4)	0.041 (3)	0.016 (3)	0.004 (3)	0.008 (3)
C31	0.066 (3)	0.074 (4)	0.079 (4)	−0.009 (3)	−0.014 (3)	0.001 (4)
C32	0.065 (3)	0.064 (4)	0.153 (6)	0.014 (3)	−0.022 (4)	−0.005 (4)
C33	0.056 (3)	0.040 (3)	0.064 (3)	−0.004 (2)	0.006 (2)	−0.006 (3)
C34	0.107 (4)	0.046 (3)	0.093 (4)	−0.001 (3)	0.018 (4)	−0.025 (3)
O1	0.078 (2)	0.054 (2)	0.055 (2)	0.0185 (19)	−0.0040 (17)	0.0052 (17)
O2	0.109 (3)	0.179 (5)	0.097 (3)	0.039 (3)	−0.026 (3)	0.022 (3)
O3	0.0630 (18)	0.0353 (18)	0.0435 (16)	0.0022 (15)	−0.0035 (14)	−0.0104 (14)
O4	0.108 (3)	0.049 (2)	0.078 (3)	0.013 (2)	−0.027 (2)	0.005 (2)

### Geometric parameters (Å, °)

**Table:**

Br1—C29	1.943 (6)	C17—C28	1.550 (6)
C1—C2	1.537 (5)	C17—C18	1.559 (5)
C1—C10	1.539 (5)	C18—C19	1.530 (5)
C1—H1A	0.9700	C18—H18	0.9800
C1—H1B	0.9700	C19—C20	1.522 (6)
C2—C3	1.504 (6)	C19—C21	1.563 (6)
C2—H2A	0.9700	C19—H19	0.9800
C2—H2B	0.9700	C20—C30	1.320 (6)
C3—O3	1.464 (5)	C20—C29	1.473 (7)
C3—C4	1.542 (6)	C21—C22	1.540 (7)
C3—H3	0.9800	C21—H21A	0.9700
C4—C24	1.534 (5)	C21—H21B	0.9700
C4—C23	1.544 (5)	C22—H22A	0.9700
C4—C5	1.563 (5)	C22—H22B	0.9700
C5—C6	1.546 (5)	C23—H23A	0.9600
C5—C10	1.551 (5)	C23—H23B	0.9600
C5—H5	0.9800	C23—H23C	0.9600
C6—C7	1.531 (6)	C24—H24A	0.9600
C6—H6A	0.9700	C24—H24B	0.9600
C6—H6B	0.9700	C24—H24C	0.9600
C7—C8	1.532 (5)	C25—H25A	0.9600
C7—H7A	0.9700	C25—H25B	0.9600
C7—H7B	0.9700	C25—H25C	0.9600
C8—C26	1.559 (5)	C26—H26A	0.9600
C8—C9	1.574 (5)	C26—H26B	0.9600
C8—C14	1.600 (5)	C26—H26C	0.9600
C9—C11	1.525 (5)	C27—H27A	0.9600
C9—C10	1.588 (5)	C27—H27B	0.9600
C9—H9	0.9800	C27—H27C	0.9600
C10—C25	1.550 (5)	C28—O1	1.431 (5)
C11—C12	1.537 (5)	C28—H28A	0.9700
C11—H11A	0.9700	C28—H28B	0.9700
C11—H11B	0.9700	C29—H29A	0.9700
C12—C13	1.526 (5)	C29—H29B	0.9700
C12—H12A	0.9700	C30—H30A	0.9300
C12—H12B	0.9700	C30—H30B	0.9300
C13—C18	1.540 (5)	C31—O2	1.211 (7)
C13—C14	1.570 (5)	C31—O1	1.313 (6)
C13—H13	0.9800	C31—C32	1.504 (8)
C14—C15	1.558 (5)	C32—H32A	0.9600
C14—C27	1.565 (5)	C32—H32B	0.9600
C15—C16	1.541 (5)	C32—H32C	0.9600
C15—H15A	0.9700	C33—O4	1.213 (6)
C15—H15B	0.9700	C33—O3	1.348 (6)
C16—C17	1.516 (6)	C33—C34	1.477 (7)
C16—H16A	0.9700	C34—H34A	0.9600
C16—H16B	0.9700	C34—H34B	0.9600
C17—C22	1.536 (6)	C34—H34C	0.9600
			
C2—C1—C10	113.4 (3)	C16—C17—C28	107.3 (4)
C2—C1—H1A	108.9	C22—C17—C28	109.6 (4)
C10—C1—H1A	108.9	C16—C17—C18	106.4 (3)
C2—C1—H1B	108.9	C22—C17—C18	101.1 (3)
C10—C1—H1B	108.9	C28—C17—C18	116.4 (3)
H1A—C1—H1B	107.7	C19—C18—C13	120.5 (3)
C3—C2—C1	109.9 (3)	C19—C18—C17	106.0 (3)
C3—C2—H2A	109.7	C13—C18—C17	111.6 (3)
C1—C2—H2A	109.7	C19—C18—H18	105.9
C3—C2—H2B	109.7	C13—C18—H18	105.9
C1—C2—H2B	109.7	C17—C18—H18	105.9
H2A—C2—H2B	108.2	C20—C19—C18	114.7 (3)
O3—C3—C2	109.8 (3)	C20—C19—C21	112.7 (3)
O3—C3—C4	107.9 (3)	C18—C19—C21	103.3 (3)
C2—C3—C4	113.6 (3)	C20—C19—H19	108.6
O3—C3—H3	108.5	C18—C19—H19	108.6
C2—C3—H3	108.5	C21—C19—H19	108.6
C4—C3—H3	108.5	C30—C20—C29	118.1 (5)
C24—C4—C3	112.0 (3)	C30—C20—C19	123.9 (4)
C24—C4—C23	108.1 (3)	C29—C20—C19	118.0 (5)
C3—C4—C23	106.9 (3)	C22—C21—C19	107.5 (4)
C24—C4—C5	115.1 (3)	C22—C21—H21A	110.2
C3—C4—C5	105.8 (3)	C19—C21—H21A	110.2
C23—C4—C5	108.8 (3)	C22—C21—H21B	110.2
C6—C5—C10	110.1 (3)	C19—C21—H21B	110.2
C6—C5—C4	114.3 (3)	H21A—C21—H21B	108.5
C10—C5—C4	117.7 (3)	C17—C22—C21	105.9 (4)
C6—C5—H5	104.4	C17—C22—H22A	110.6
C10—C5—H5	104.4	C21—C22—H22A	110.6
C4—C5—H5	104.4	C17—C22—H22B	110.6
C7—C6—C5	110.5 (3)	C21—C22—H22B	110.6
C7—C6—H6A	109.6	H22A—C22—H22B	108.7
C5—C6—H6A	109.6	C4—C23—H23A	109.5
C7—C6—H6B	109.6	C4—C23—H23B	109.5
C5—C6—H6B	109.6	H23A—C23—H23B	109.5
H6A—C6—H6B	108.1	C4—C23—H23C	109.5
C6—C7—C8	113.7 (3)	H23A—C23—H23C	109.5
C6—C7—H7A	108.8	H23B—C23—H23C	109.5
C8—C7—H7A	108.8	C4—C24—H24A	109.5
C6—C7—H7B	108.8	C4—C24—H24B	109.5
C8—C7—H7B	108.8	H24A—C24—H24B	109.5
H7A—C7—H7B	107.7	C4—C24—H24C	109.5
C7—C8—C26	106.8 (3)	H24A—C24—H24C	109.5
C7—C8—C9	109.5 (3)	H24B—C24—H24C	109.5
C26—C8—C9	111.9 (3)	C10—C25—H25A	109.5
C7—C8—C14	110.5 (3)	C10—C25—H25B	109.5
C26—C8—C14	109.2 (3)	H25A—C25—H25B	109.5
C9—C8—C14	109.0 (3)	C10—C25—H25C	109.5
C11—C9—C8	110.4 (3)	H25A—C25—H25C	109.5
C11—C9—C10	114.8 (3)	H25B—C25—H25C	109.5
C8—C9—C10	116.5 (3)	C8—C26—H26A	109.5
C11—C9—H9	104.5	C8—C26—H26B	109.5
C8—C9—H9	104.5	H26A—C26—H26B	109.5
C10—C9—H9	104.5	C8—C26—H26C	109.5
C1—C10—C25	107.8 (3)	H26A—C26—H26C	109.5
C1—C10—C5	107.7 (3)	H26B—C26—H26C	109.5
C25—C10—C5	114.1 (3)	C14—C27—H27A	109.5
C1—C10—C9	108.3 (3)	C14—C27—H27B	109.5
C25—C10—C9	112.4 (3)	H27A—C27—H27B	109.5
C5—C10—C9	106.4 (3)	C14—C27—H27C	109.5
C9—C11—C12	113.4 (3)	H27A—C27—H27C	109.5
C9—C11—H11A	108.9	H27B—C27—H27C	109.5
C12—C11—H11A	108.9	O1—C28—C17	110.7 (3)
C9—C11—H11B	108.9	O1—C28—H28A	109.5
C12—C11—H11B	108.9	C17—C28—H28A	109.5
H11A—C11—H11B	107.7	O1—C28—H28B	109.5
C13—C12—C11	112.4 (3)	C17—C28—H28B	109.5
C13—C12—H12A	109.1	H28A—C28—H28B	108.1
C11—C12—H12A	109.1	C20—C29—Br1	112.4 (4)
C13—C12—H12B	109.1	C20—C29—H29A	109.1
C11—C12—H12B	109.1	Br1—C29—H29A	109.1
H12A—C12—H12B	107.9	C20—C29—H29B	109.1
C12—C13—C18	116.0 (3)	Br1—C29—H29B	109.1
C12—C13—C14	110.6 (3)	H29A—C29—H29B	107.9
C18—C13—C14	109.9 (3)	C20—C30—H30A	120.0
C12—C13—H13	106.6	C20—C30—H30B	120.0
C18—C13—H13	106.6	H30A—C30—H30B	120.0
C14—C13—H13	106.6	O2—C31—O1	121.8 (6)
C15—C14—C27	107.6 (3)	O2—C31—C32	124.4 (6)
C15—C14—C13	110.5 (3)	O1—C31—C32	113.7 (6)
C27—C14—C13	109.6 (3)	C31—C32—H32A	109.5
C15—C14—C8	110.6 (3)	C31—C32—H32B	109.5
C27—C14—C8	110.1 (3)	H32A—C32—H32B	109.5
C13—C14—C8	108.5 (3)	C31—C32—H32C	109.5
C16—C15—C14	115.1 (3)	H32A—C32—H32C	109.5
C16—C15—H15A	108.5	H32B—C32—H32C	109.5
C14—C15—H15A	108.5	O4—C33—O3	123.4 (4)
C16—C15—H15B	108.5	O4—C33—C34	124.9 (5)
C14—C15—H15B	108.5	O3—C33—C34	111.7 (5)
H15A—C15—H15B	107.5	C33—C34—H34A	109.5
C17—C16—C15	112.6 (3)	C33—C34—H34B	109.5
C17—C16—H16A	109.1	H34A—C34—H34B	109.5
C15—C16—H16A	109.1	C33—C34—H34C	109.5
C17—C16—H16B	109.1	H34A—C34—H34C	109.5
C15—C16—H16B	109.1	H34B—C34—H34C	109.5
H16A—C16—H16B	107.8	C31—O1—C28	117.5 (4)
C16—C17—C22	116.2 (3)	C33—O3—C3	118.4 (3)

### Hydrogen-bond geometry (Å, °)

**Table:**

*D*—H···*A*	*D*—H	H···*A*	*D*···*A*	*D*—H···*A*
C32—H32A···O4^i^	0.96	2.48	3.365 (7)	154
C28—H28B···O4^ii^	0.97	2.57	3.487 (6)	158

Symmetry codes: (i) *x*+1, *y*−1, *z*; (ii) *x*, *y*−1, *z*.
